# Reviewer acknowledgement 2012

**DOI:** 10.1186/1471-2180-13-21

**Published:** 2013-02-19

**Authors:** Philippa K Harris

**Affiliations:** 1 BioMed Central, 236 Gray’s Inn, Road, London, WC1X 8HB, UK

## Abstract

**Contributing reviewers:**

The editors of *BMC Microbiology* would like to thank all our reviewers who have contributed to the journal in Volume 12 (2012).

## 

Pervaiz Abbasi

Canada

Mohamed Abouelhoda

Egypt

Menandro Acda

Philippines

Hans-Wolfgang Ackermann

UK

Lorenz Adrian

Germany

Laura Alcazar Fuoli

Spain

Herve Alexandre

France

Merima Alispahic

Austria

Heather Allen

USA

Sandro Almeida

Brazil

Covadonga Alonso

Spain

Scott Alper

USA

Christopher Alteri

USA

Burim Ametaj

Canada

Antonella Amicucci

Italy

Robin Anderson

USA

Siv Andersson

Sweden

Jan Andersson

Sweden

Vincenza Andreoni

Italy

Fernando Andreote

Brazil

Emmanuel Andres

France

Oystein Angen

Denmark

Esther Angert

USA

Florent Angly

Australia

Wu Anhua

China

Andrey Anisimov

Russia

Dionysios Antonopoulos

USA

Charles Apperson

USA

Shree Kumar Apte

India

Virginia Aragon

Spain

Marie Archambault

Canada

Fabrice Armougom

France

Bernard Arulanandam

USA

Lena Aslund

Sweden

Matias Attene Ramos

USA

Ramy Attia

USA

David AuCoin

USA

Marcela Audisio

Argentina

Diana Axelsson Olsson

Sweden

Juan Ayala

Spain

Stéphane Aymerich

France

Vasco Azevedo

Brazil

Ramy Karam Aziz

Egypt

Susan Bach

Canada

Martin Backor

Slovakia

Charles Bacon

USA

Charles Baer

USA

Fabio Bagnoli

Italy

Mohammed Bahey-El-Din

Egypt

Martin Iain Bahl

Denmark

Krishnaswamy Balamurugan

India

Laura Baldoma

Spain

Mitchell Balish

USA

Livia Balsalobre

Brazil

Julia Bandow

Germany

Fernando Barcellos

Brazil

Francisco Barona-Gomez

Mexico

Joyoti Basu

India

Ken Bayles

USA

Chris Bayliss

UK

Bernard Beall

USA

Stephen Bearne

Canada

Cecile Bebear

France

Arturo Becerra

Mexico

Relja Beck

Croatia

Georgios Belibasakis

Switzerland

Christophe Michel Yves Beloin

France

Jorge Benach

USA

Marlene Benchimol

Brazil

Eshel Ben-Jacob

Israel

Maurice Bennink

USA

Stephen Beres

USA

Indra Bergval

Netherlands

Chrystal Berry

Canada

Francesco Berti

Italy

Bhaskar Bhadra

India

Giancarlo Biagini

UK

Jennifer Biddle

USA

Tomasz Bielecki

Poland

Franck Biet

France

Wolfgang Bilger

Germany

Harry Birkbeck

UK

Markus Bischoff

Germany

Wilbert Bitter

Netherlands

Jason Blackard

USA

Leila Blackman

Australia

Alain Blanchard

France

Jorge Blanco

Spain

Carlos Blanco

France

Sophie Bleves

France

James Bliska

USA

Pierre Bogaerts

Belgium

Erwin Bohn

Germany

Ivo Boneca

France

Mariangela Bonizzoni

USA

Nagamani Bora

UK

Thomas Boren

Sweden

Giuseppe Botta

Italy

Peter Bottomley

USA

Charles Boucher

Netherlands

Philippe Bouloc

France

Dawn Bowdish

Canada

Jolene Bowers

USA

Jessica Boyd

Nigeria

Rosie Bradshaw

New Zealand

Rogelio Brandao

Brazil

Adriano Brandelli

Brazil

Trygve Brautaset

Norway

Stéphane Bretagne

France

Yves Briers

Belgium

Sylvain Brisse

France

Susana Brom

Mexico

Jeremy Brown

UK

Daniel Brown

USA

Steven Brown

USA

Reinhold Brückner

Germany

Dunja Bruder

Germany

Stanley Brul

Netherlands

Jorg Brunner

Netherlands

Harald Brussow

Switzerland

Carmen Buchrieser

France

Bryce Buddle

New Zealand

Girbe Buist

Netherlands

Tim Bull

UK

David Burke

USA

Víctor Bustamante

Mexico

Geraldine Butler

Ireland

Pietro Buzzini

Italy

Thomas Byrd

USA

Simone Mario Caccio'

Italy

Gustavo Caetano-Anolles

USA

Michael Calcutt

USA

Todd Callaway

USA

Maria Luisa Callegari

Italy

Edmundo Calva

Mexico

Caroline Cameron

Canada

Floriana Campanile

Italy

Kenneth Campellone

USA

Gerard Cangelosi

USA

Manuela Caniça

Portugal

Maria Isabel Nogueira Cano

Brazil

David Canovas

Spain

Xudong Cao

Canada

Nicholas Carbonetti

USA

Maria Angelica Cardoso

Brazil

Magnus Carlquist

Sweden

Catherine Carrillo

Canada

Eric Cascales

France

Mariana Castanheira

USA

Angel Cataldi

Argentina

Shaun Cawthraw

UK

Marina Cerquetti

Italy

Florian Chain

France

Gopal Chakrabarti

India

Pradip Chakraborti

India

Stephen Challacombe

UK

Patricia Champion

USA

Sheela Chandra

India

Wei-Lun Chang

Taiwan

Narisara Chantratita

Thailand

Wei-Liang Chao

Taiwan

Trevor Charles

Canada

Emmanuelle Charpentier

Sweden

Roy Chaudhuri

UK

Franck Chauvat

France

Yi Chen

USA

Chen Chen

China

Ming-Ju Chen

Taiwan

Jeffrey Chen

Switzerland

Wangxue Chen

Canada

Guang-Wu Chen

Taiwan

Erica Chimara

Brazil

Luciane Chimetto

Brazil

Tae-Ju Cho

South Korea

Nam-Hyuk Cho

South Korea

Yen-Hung Chow

Taiwan

Ryszard Chróst

Poland

Thomas Chrzanowski

USA

Maurizio Ciani

Italy

Daniela M Cirillo

Italy

Thomas Clarke

UK

Christopher Clarke

USA

Robert Click

USA

Axel Cloeckaert

France

Martha Clokie

UK

Muriel Cocaign-Bousquet

France

Marita Cohn

Sweden

Nick Coldham

UK

Garry Cole

USA

James Cole

USA

Carlos Colinas

Spain

Maria Carmen Collado

Spain

Justine Collier

Switzerland

Mattias Collin

Sweden

Desmond Collins

New Zealand

Elena Maria Comelli

Canada

L Connell

USA

Ian Connerton

UK

Geoffrey Coombs

Australia

Stuart Cordwell

Australia

Pierre Cornelis

Belgium

Vitor Costa

Portugal

Paul Cotter

Ireland

B. Cournoyer

France

Elena Crotti

Italy

Christopher Crowder

USA

Jaime Cubero

Spain

Narcisa Cunha-e-Silva

Brazil

Elisa Cupolillo

Brazil

Juliano Cury

Brazil

Jungui Dai

China

Elis Dalla Costa

Brazil

Richard Darveau

USA

Rupali Datta

USA

Vincent Daubin

France

Rimantas Daugelavicius

Lithuania

Maria De Angelis

Italy

Carlotta De Filippo

Italy

Donatella de Pascale

Italy

Nathalie Declerck

France

Tom Defoirdt

Belgium

Céline Delbes-Paus

France

franco dellaglio

Italy

Tanneke den Blaauwen

Netherlands

Jonathan Dennis

Canada

Rajendar Deora

USA

Amare Deribew

Ethiopia

Mickael Desvaux

France

Marc Devocelle

Ireland

Subramanian Dhandayuthapani

USA

Irene Dige

Denmark

Elizabeth Dinsdale

USA

Benoit Divol

South Africa

Douglas Dixon

USA

Julianne Djordjevic

Australia

Thuy Do

UK

Ulrich Dobrindt

Germany

Monika Dolejska

Czech Republic

M. Angeles Dominguez

Spain

stefano donadio

Italy

Justin Donato

USA

Giovanna Donnarumma

Italy

Marina Donova

Russia

David Donovan

USA

Horng-Yunn Dou

Taiwan

Shaynoor Dramsi

France

Michel Drancourt

France

David Dubnau

USA

Eugenie Dubnau

USA

Edward Dudley

USA

Alain Dufour

France

Marie Dufresne

France

Guillaume Dumenil

France

Micah Dunthorn

Germany

Sylvain Durand

France

Jonathan Dworkin

USA

Andrew Edwards

UK

Thomas Egli

Switzerland

Garth Ehrlich

USA

Zehava Eichenbaum

USA

Dalèle Elhani

Tunisia

Craig Ellermeier

USA

Matthew Ellison

USA

Walid El-Sharoud

Egypt

Susanne Engelmann

Germany

Harold Erickson

USA

Klaus Eschrich

Germany

Jaime Esteban

Spain

Gilles Etienne

France

Chad Euler

USA

Thomas Evans

UK

Paul Everest

UK

Alan Fahey

Ireland

Matteo Falasconi

Italy

Juliana Pfrimer Falcao

Brazil

Ana Falcon

Spain

Gang Fang

USA

Mauricio Farfan

Chile

Eric Faudry

France

Maryse Fauville

Belgium

J. Christopher Fenno

USA

Jean-Baptiste Ferdy

France

Pilar Fernandez-Ibañez

Spain

Eugenio Ferrari

Italy

Joseli Ferreira

Brazil

James Ferry

USA

Paul Fey

USA

Thomas Ficht

USA

Patrick Fickers

Belgium

Agnes Figueiredo

Brazil

Antonio Figueras

Spain

Melanie Filiatrault

USA

Djordje Fira

Serbia

Wolfgang Fischer

Germany

Michael Flythe

USA

Marco Fondi

Italy

Katrina Forest

USA

Christiane Forestier

France

Florence Forget-Richard

France

Ake Forsberg

Sweden

Simon Foster

UK

Kevin Foster

UK

Betsy Foxman

USA

Rennos Fragkoudis

UK

Jose Franco da Silveira

Brazil

Alexander Franz

USA

Maria Fredriksson-Ahomaa

Finland

Stephanie Friedman

USA

Jens Frisvad

Denmark

Zhen Fu

USA

Pio Maria Furneri

Italy

Julio Galvez

Spain

Yunn-Hwen Gan

Singapore

George Gao

China

He Gao

China

Haichun Gao

China

Dea Garcia Hermoso

France

Emilia Garcia Moruno

Italy

Eleonora García Véscovi

Argentina

Jose F Garcia-Mazcorro

Mexico

Jaime García-Mena

Mexico

Santi Garcia-Vallve

Spain

Sonia Garde

Spain

Richard Gardner

New Zealand

Carlo Garzelli

Italy

Pep Gasol

Spain

Susanne Gebhard

Germany

Nelson Gekara

Sweden

Tesfaye Gelanew

USA

Joan Geoghegan

Ireland

Thomas Gerken

USA

Giovanni Gherardi

Italy

Michel Ghislain

Belgium

Giuseppe Giammanco

Italy

Tommaso Giani

Italy

Thierry Giardina

France

Rafal Gierczynski

Poland

Steven Gill

USA

Brent Gilpin

New Zealand

Philippe Glaser

France

Richard Goering

USA

Suely Gomes

Brazil

Tania Gomes

Brazil

Bruno Gomez-Gil

Mexico

Paulino Gomez-Puertas

Spain

Juan Gonzalez

USA

Ramón González

Spain

Jose Eduardo Gonzalez-Pastor

Spain

Steven Goodman

USA

Andrew Gorringe

UK

Masatoshi Goto

Japan

Marcelo Gottschalk

Canada

Friedrich Götz

Germany

Mark Goulian

USA

Joerg Graf

USA

Gregor Grass

Germany

Jeff Green

UK

Miriam Greenberg

USA

Gustavo Bueno Gregoracci

Brazil

Gareth Griffiths

Germany

Rob Griffiths

UK

Gideon Grogan

UK

Joyef Grones

Slovakia

Michael Grusak

USA

Ioannis Gryllos

USA

Bjarnheidur Gudmundsdottir

Iceland

Julie Guignot-degenis

France

Adam Guss

USA

Maximiliano Gutierrez

Germany

Julian Guttman

Canada

Pieter-Jan Haas

Netherlands

Abderrahman Hachani

UK

Susan Hagen

USA

Matthias Hahn

Germany

Mohamed-Ali Hakimi

France

Roy Hall

Australia

Patrick Hallenbeck

Canada

Karin Hammer

Denmark

Leendert Hamoen

UK

Yanping Han

China

Lynn Hancock

USA

Robert Hancock

Canada

Scott Handley

USA

Hilde Hansen

Norway

Emanuel Hanski

Israel

Brendon Hanson

Singapore

Klaus Hantke

Germany

Heather Harbottle

USA

Kusum Harjai

India

Freya Harrison

UK

Anton Hartmann

Germany

John Hartung

USA

Colin Harwood

UK

Zahra Hasan

Pakistan

Henrik Hasman

Denmark

Graham Hatfull

USA

David Hayman

USA

Shaomei He

USA

Ralf Heermann

Germany

David Heinrichs

Canada

Sophie Helaine

UK

John Helmann

USA

Birgit Henrich

Germany

Laura Herrera-Leon

Spain

Erik Hewlett

USA

Janet Hill

Canada

Stuart Hill

USA

Karen Hill

USA

Deborah Hinton

USA

Zakaria Hmama

Canada

Ayala Hochman

Israel

Paul Hoffman

USA

Mark Holmes

UK

Kathryn Holt

Australia

Masafumi Horie

Japan

Jun-ichi Horiuchi

Japan

Alexander Horswill

USA

Paul Horwood

Papua New Guinea

Hans-Peter Horz

Germany

Wen-Chi Hou

Taiwan

Kurt Houf

Belgium

Gordon Howarth

Australia

Ben Howden

Australia

Chieh-Chen Huang

Taiwan

Xinxiang Huang

China

Kaiyao Huang

China

Bernhard Hube

Germany

Melanie Huch

Germany

Laura Hug

Canada

Diarmaid Hughes

Sweden

Cornelis J. J. Huijsmans

Netherlands

Romney Humphries

USA

Sabine Hunke

Germany

Javid Hussain

Oman

Mark Ibekwe

USA

Valerio Iebba

Italy

Kunio Ihara

Japan

Juan Imperial

Spain

Heribert Insam

Austria

Toshikazu Irie

Japan

RE Isaacson

USA

Teruyo Ito

Japan

Tomotada Iwamoto

Japan

Andrew Paul Jackson

UK

Marie-Agnès Jacques

France

Nick Jakubovics

UK

Euan James

UK

Tong-Rong Jan

Taiwan

Ludwig Jardillier

France

Aftab Jasir

Sweden

Mohamed Jebbar

France

Dieter Jendrossek

Germany

Slade Jensen

Australia

Susan Jensen

Canada

Jorgen Skov Jensen

Denmark

Che Ok Jeon

South Korea

Mark Jepson

UK

Xin-Ming Jia

China

Antonio Jimenez

Spain

Antonio Jiménez-Ruiz

Spain

Meilin Jin

China

Shouguang Jin

USA

Gerwald Jogl

USA

Reid Johnson

USA

Sheena Johnson

UK

Timothy Johnson

USA

Stuart Jones

USA

Anne Katherine Jones

USA

Kathryn Jones

USA

Christine Josenhans

Germany

Juan Luis Jurat-Fuentes

USA

Tsutomu Kakuda

Japan

Neville Kallenbach

USA

Susan Kaminskyj

Canada

C. Cheng Kao

USA

Zeynep Ceren Karahan

Turkey

Matti Karp

Finland

Balakrishnan Karuppiah

India

Makoto Kawamukai

Japan

Kiyoshi Kawasaki

Japan

Jacqui Keenan

New Zealand

William Kelly

New Zealand

Robert Kelly

USA

Linda Kelly

USA

Judit Szilvia Keseru

Hungary

Nemat Keyhani

USA

Asad Khan

India

Stephen Kidd

Australia

Yoshitomo Kikuchi

Japan

Abdullah Kilic

Turkey

Dong Wook Kim

South Korea

Younghoon Kim

South Korea

Kristen Kindrachuk

USA

Samantha King

USA

Ioannis Kioumis

Greece

Andre Kipnis

Brazil

Theo Kirkland

USA

Leif Kirsebom

Sweden

Lisa Klasson

Sweden

Laura Klepp

Argentina

MIchael Klocke

Germany

Wen-Chien Ko

Taiwan

Ichizo Kobayashi

Japan

Marleen Kock

South Africa

Ali Kocyigit

Turkey

Ralf Kölling

Germany

Jian Kong

China

Rok Kopinc

Slovenia

Agata Krawczyk-Balska

Poland

Ricardo Kruger

Brazil

Claude Krummenacher

USA

Katrin Kuhls

Germany

Suresh Kumar

India

Apurba Kumar Sau

India

Muthiah Kumaraswami

USA

Howard Kuramitsu

USA

Elizabeth Kutter

USA

Dong Kwon

USA

Hartmut Laatsch

Germany

Maurizio Labbate

Australia

Elisabeth Labruyere

France

Charles Lacey

UK

Michaela Lackner

Austria

Marc Lacroix

Belgium

Marcelo Laia

Brazil

Gyanu Lamichhane

USA

Richard Lamont

USA

Lefu Lan

China

Paolo Landini

Italy

Chernousova Larisa

Russia

Marie-Frederique Lartigue

France

Christine Lascols

USA

Elena Lasunskaia

Brazil

Stanley Lau

Hong Kong

Andrea Laukova

Slovakia

Sylvia Leao

Brazil

Francois Lebreton

USA

Peter Lee

Japan

Sangmi Lee

USA

Laura Leff

USA

Michael Lehman

USA

Lars I Leichert

Germany

Jose Lemos

USA

Livia Leoni

Italy

Andrey Letarov

Russia

Bruno Levecke

Belgium

Bruce Levin

USA

Shawn Lewenza

Qatar

Astrid Lewin

Germany

Xian-Zhi Li

Canada

Robert Li

USA

Claude Libert

Belgium

Mark Liles

USA

Shuangjun Lin

China

Thomas Lion

Austria

Zhiqiang Liu

China

Changting Liu

China

George Liu

USA

Karen G Lloyd

Denmark

Durga Srinivas Lodagala

India

Bettina Löffler

Germany

Gunnar Loh

Germany

John Lopes

USA

Cesar Lopez Camarillo

Mexico

Graciela Lorca

USA

Francoise Lucas

France

Tillmann Lueders

Germany

Joe Lutkenhaus

USA

Daniel M Linares

Spain

Zhonghua Ma

China

Wenbo Ma

USA

Kesen Ma

Canada

Simone Maccaferri

Italy

Jose Machado

Portugal

Janet MacInnes

Canada

Michelle Maclean

UK

Robert Madden

UK

Frank Madeo

Austria

Walter Magliani

Italy

Taifo Mahmud

USA

Tomas Maira-Litran

USA

Pawan Malhotra

India

Johan Malmstrom

Sweden

Georgina Manning

UK

Matteo Marangon

Australia

Hélène Marchandin

France

Maria Marco

USA

Richard Marconi

USA

Gregory Marczynski

Canada

Anthony Maresso

USA

Imma Margarit

Italy

Gergely Maroti

Hungary

Philip Marsh

UK

Eric Martens

USA

Laura Martin

Italy

Susana Martin-Orue

Spain

Flaviano Martins

Brazil

Hideaki Maseda

Japan

Vega Masignani

Italy

Rosalind Maskell

UK

Rob Massung

USA

Juan Mata

UK

Luis Mateos

Saint Pierre and Miquelon

Pedro F. Mateos

Spain

Sohkichi Matsumoto

Japan

James Matsunaga

USA

Shigenobu Matsuzaki

Japan

Michael Matthias

USA

Thithiwat May

USA

Luca Mazzon

Italy

Mark McClain

USA

Glenn McConkey

UK

Richard McCulloch

UK

Matthew McCusker

Ireland

Christopher McDevitt

Australia

John McDonald

USA

Lesley McGee

USA

Chris McGowin

USA

Kevin McGuigan

Ireland

Robert McLean

USA

David McMillen

Canada

Alan McNally

UK

Michael McNeil

USA

Friedhelm Meinhardt

Germany

Jay Mellies

USA

Mariza Melo

Brazil

Kristina Mena

USA

Armelle Ménard

France

Jairo Mendez

Colombia

Regis Mendonca

Chile

Guoyu Meng

China

Dominique Mengin-Lecreulx

France

Alessio Mengoni

Italy

Max Mergeay

Belgium

Andres Merits

Estonia

Kaixia Mi

China

Jan Michiels

Belgium

Jonathan Mielenz

USA

William Miller

USA

Dan Miller

USA

M Miragaia

Portugal

Kildare Miranda

Brazil

Raghavendra Mirmira

USA

Norihiko Misawa

Japan

Nidhi Mishra

India

Tim Mitchell

UK

Stefano Mocali

Italy

Petra Moebius

Germany

Debasisa Mohanty

India

Ghasemali Mohebali

Iran

Eiman Mokaddas

Kuwait

Igor Mokrousov

Russia

Douwe Molenaar

Netherlands

Kuvat Momynaliev

Russian Federation

Stefan Monecke

Germany

Paul Monis

Australia

Hans-Jurg Monstein

Sweden

Frits Mooi

Netherlands

Margo Moore

Canada

Melanie Mormile

USA

Robert Morris

USA

Daniel Morton

USA

Monica Moschioni

Italy

Samuel Moskowitz

USA

Serge Mostowy

France

Richard Moxon

UK

Martin Muller

Germany

Matthew Mulvey

USA

Timothy Murphy

USA

Sean Murray

USA

Thomas Murray

USA

Heath Murray

UK

Sean Murray

USA

James Musser

USA

Guenther Muth

Germany

Rahul Nair

Singapore

Noriko Nakajima

Japan

Beiyan Nan

USA

Tonny Naranjo

Colombia

Denise Nardelli-Haefliger

Switzerland

Andrea Nascimento

Brazil

Ana Lucia Nascimento

Brazil

Andrea Nascimento

Brazil

Gerardo Nava

USA

William Navarre

USA

Fernando Navarro-Garcia

Mexico

Prasanna Neelakantan

India

Natasha Nesbitt

USA

Christophe Nguyen-The

France

Kendra Nightingale

USA

Anastasia Nijnik

Canada

Michele Nishiguchi

USA

Yoshikazu Nishikawa

Japan

Yorihiro Nishimura

Japan

Mikkel Nissum

Italy

Hideaki Nojiri

Japan

Francoise Norel

France

Agnieszka Nowak

Poland

Marisol Ocampo

Colombia

James O'Gara

Ireland

Tae-Jin Oh

South Korea

D. Elizabeth O'Hanlon

USA

Iwao Ojima

USA

Mónica Oleastro

Portugal

Richard Oliver

Australia

Javier Ollero

Spain

Jonathan Olson

USA

Ken Olson

USA

Rene Olsthoorn

Netherlands

Ertan Emek Onuk

Turkey

Diana Oram

USA

Katarina Oravcova

UK

Diane Ordway

USA

Michael Otto

USA

Hong-Yu Ou

China

Omar Oyarzabal

USA

Oleg Paliy

USA

Mark Pallen

UK

Kelli Palmer

USA

Stefan Panaiotov

Bulgaria

Bo Pang

China

Gloria Luz Paniagua

Mexico

Barbara Papadopoulou

Canada

Craig Parker

USA

Ben Pascoe

UK

Darshankumar Pathak

USA

Sheila Patrick

UK

David Peabody

USA

Mike Peck

UK

Alvaro Peix

Spain

Xuanxian Peng

China

András Penyige

Hungary

Alan Perelson

USA

Antonio J. Pérez-Pulido

Spain

Georg Peters

Germany

Jeannine Petersen

USA

Stephen Peterson

USA

Marie-Agnès Petit

France

Maria Julia Pettinari

Argentina

Stacy Pfaller

USA

Ilona Pfeiffer

Hungary

Sangita Phadtare

USA

Mathieu Picardeau

France

Gerald Pier

USA

Ellen Pierce

USA

Gerhard Pietersen

South Africa

Gorben Pijlman

Netherlands

Martin Pilhofer

USA

Paola Pilo

Switzerland

Madalena Pimentel

Portugal

Joanne Platell

Australia

Patrick Plesiat

France

Jan Poolman

Netherlands

David Popham

USA

Yannick Poquet

France

Andrea Porras-Alfaro

USA

Jan Potempa

USA

Nicola Pozzato

Italy

Balaji Prakash

India

Judy Praszkier

Australia

Peter Preiser

Singapore

Morgan Price

USA

Richard Proctor

USA

Daniele Provenzano

USA

Xudong Qu

China

Dulciene Queiroz

Brazil

Enrique Quesada Moraga

Spain

Janet Quinn

UK

Noura Raddadi

Italy

Maria Isabel Ramos-Gonzalez

Spain

Reuben Ramphal

France

Kalliopi Rantsiou

Italy

Vicki Rapp Gabrielson

USA

Madeleine Ravaoarinoro

Canada

Jacques Ravel

USA

Manickam Ravichandran

Malaysia

Mamta Rawat

USA

Debabrata Ray Chaudhuri

USA

Giuseppina Rea

Italy

Lúcia Rebello Dillenburg

Brazil

Dominik Refardt

Switzerland

Gregor Reid

Canada

Joachim Reidl

Austria

Michael Reith

Canada

Han Remaut

Belgium

Dacheng Ren

USA

Gregory Resch

Switzerland

Mark Reuter

UK

Sylvie Reverchon

France

Peter Revill

Australia

Ryan Rhodes

USA

Marcelo Ribeiro

Brazil

Ezio Ricca

Italy

Scott Rice

Australia

Volker Rickerts

Germany

Christian Riedel

Germany

Kristian Riesbeck

Sweden

Lee Riley

USA

Margaret Riley

USA

Tamar Ringel-Kulka

USA

Deborah Roberts

Canada

Gary Roberts

USA

Kelly Robertson

USA

Ashley Robinson

USA

Tatiana Rochat

France

Juliany Cola Fernandes Rodrigues

Brazil

Pablo Rodriguez

Spain

Geraint Rogers

UK

Wilfred Roling

Netherlands

Elvira Román

South Georgia and the South Sandwich Is

Sara Romano

France

Eliete Romero

Brazil

Simona Rondini

Italy

Clive Ronson

New Zealand

Gail Rosen

USA

Maria Lucia Rosa Rossetti

Brazil

Michael Rothballer

Germany

Michae l Rother

Germany

Bart Roucourt

Belgium

Joel Rudney

USA

Natividad Ruiz

USA

Estella Ruiz Baca

Mexico

Michael Rust

USA

Jan Ruzicka

Czech Republic

Maurizio Ruzzi

Italy

Elizabeth Ryan

USA

Sangryeol Ryu

South Korea

Orhan Sahin

USA

Milton Saier

USA

Shilpakala Sainath Rao

USA

Umadevi Sajjan

USA

Seema Saksena

USA

Olga Sakwinska

Switzerland

Jean-Michel Sallenave

France

Vittorio Sambri

Italy

Elizabeth Sampaio

Brazil

Nicole Sampson

USA

James Samuel

USA

Scott Samuels

USA

Yolanda Sanchez

Spain

Juan Sanjuan

Spain

Marìa de la Paz Santangelo

Argentina

Marina Santic

Croatia

Jorge Santo Domingo

USA

Renato Santos

Brazil

Ilda Santos-Sanches

Portugal

Hugo Sarmento

Spain

Reetta Satokari

Finland

Bernadette Saunders

Australia

Sven J. Saupe

France

Kirsi Savijoki

Finland

Gary Sawers

Germany

Deepak Saxena

USA

Murat Sayan

Turkey

Vincenzo Scarlato

Italy

Mark Schembri

Australia

Ruth Schmitz-Streit

Germany

Anna Schnürer

Sweden

Jeff Schorey

USA

Catherine Schouler

France

Imke Schroeder

USA

Wolfgang Schumann

Germany

Ira Schwartz

USA

Elke Schweda

Sweden

Martin Schwemmle

Germany

Andreas Schwiertz

Germany

Kathryn Scott

UK

June Scott

USA

Bruce Seal

USA

Ondrej Sedo

Czech Republic

Lucy Seldin

Brazil

Joseph Selvin

India

Ilkin Sengün

Turkey

Angela Sessitsch

Austria

Abdul Rauf Shakoori

Pakistan

Cander Shekhar Sharma

USA

Shew Meei Sheu

Taiwan

Michael Shiloh

USA

Won-Bo Shim

USA

Jeong Hwan Shin

South Korea

Naoya Shinzato

Japan

Adebayo Shittu

Nigeria

Sisinthy Shivaji

India

Annie Shrestha

Canada

Hung-Yu Shu

Taiwan

Krzysztof Sieradzki

USA

Jörg Simon

Germany

Shoor Vir Singh

India

Leung Kei Siu

Taiwan

Anna Kaarina Sivonen

Finland

Asa Sjoling

Sweden

David Skurnik

USA

David Smajs

Czech Republic

Duncan R. Smith

Thailand

Hilde Smith

Netherlands

Lori Snyder

UK

Lotte Sogaard-Andersen

Germany

Ladislav Soják

Slovakia

Evgeni Sokurenko

USA

Glenn Songer

USA

Elena Soria Soria

Spain

Marco Soriani

Italy

Marco Soriani

Italy

Giuseppe Spano

Italy

Joachim Spergser

Austria

Patrizia Spigaglia

Italy

Lisa D. Sprague

Germany

Ranjan Srivastava

USA

Tyler St. Denis

USA

Nicola Stanley-Wall

UK

Bärbel Stecher

Germany

Gerard Stelma Jr.

USA

Julie Stenken

USA

Karen Stevenson

UK

O. Colin Stine

USA

Tim Stinear

Australia

Ellen Stobberingh

Netherlands

Jiri Stulik

Czech Republic

Jörg Stülke

Germany

Jan Suchodolski

USA

Reiko Sugiura

Japan

Sang-Jin Suh

USA

Andreas Suhrbier

Australia

Yi-Cheng Sun

China

Lotta-Riina Sundberg

Finland

Arnfinn Sundsfjord

Norway

Michael Surette

Canada

Staffan Svard

Sweden

Kerstin Svensson

Sweden

Popi Syntichaki

Greece

Florian Szabados

Germany

Steven Szczepanek

USA

Hendrik Szurmant

USA

Eduardo Taboada

Canada

Carlos Taborda

Brazil

Kiyoshi Tajima

Japan

Maria Takacs

Hungary

Shinichi Takaichi

Japan

Rodrigo Taketani

Brazil

Ben Tall

USA

Kar-Chun Tan

Australia

Julian Tanner

Hong Kong

Neslihan Tas

USA

Rohan Teasdale

Australia

George Tegos

USA

Lucia Teixeira

Brazil

Henrique Teixeira

Brazil

Nigel Ternan

UK

Connor Thomas

Australia

Skye Thomas-Hall

Australia

Fabiano Thompson

Brazil

Menaka Thounaojam

India

Cameron Thrash

USA

Richard Titball

UK

Andrew Tomaras

USA

Herbert Tomaso

Germany

Jan Tommassen

Netherlands

Eva Top

USA

Alfredo Torres

USA

Carmen Torres

Spain

Eila Torvinen

Finland

Yuzuru Tozawa

Japan

Armen Trchounian

Armenia

Marie Tre-Hardy

France

Herman Tse

Hong Kong

Ching-Ping Tseng

Taiwan

Christine Turenne

Canada

Peter Turnbaugh

USA

Kevin Tyler

UK

Glen Ulett

Australia

Jose Usme-Ciro

Colombia

Pongsak Utaisincharoen

Thailand

Manjula Vagarali

India

Dino Vaira

Italy

Bruce Vallance

Canada

Jaione Valle

Spain

Janneke van de Wijgert

Netherlands

Wil van der Reijden

Netherlands

Jan Maarten van Dijl

Netherlands

Andrewe Van Kessel

Canada

Jos van Putten

Netherlands

Willem van Schaik

Netherlands

Douwe Van Sinderen

Ireland

Guido Vanham

Belgium

Nongnuch Vanittanakom

Thailand

Pierre Vaudaux

Switzerland

Ana Isabel Vela

Spain

Iván Ventoso

Spain

Kristin Verbeke

Belgium

Gilles Vergnaud

France

Ashutosh Verma

USA

Andre Vermeglio

France

Mónica Vermeulen

Argentina

Miguel Vicente

Spain

Ana Carolina Vicente

Brazil

Miguel Vicente

Spain

Tomas Villa

Spain

Alberto Villasenor

Mexico

Gabriel Vinderola

Argentina

Marko Virta

Finland

Ulrich Vogel

Germany

Amy Vogler

USA

Uwe Völker

Germany

Atte von Wright

Finland

William Wade

UK

David M. Wagner

USA

Sun Nyunt Wai

Sweden

Steve Wakelin

New Zealand

Graham Walker

USA

Fiona Walsh

Switzerland

Caixia Wan

USA

Mei Wang

USA

Chunxia Wang

USA

Guangyi Wang

USA

Xiaoyu Wang

USA

Chengshu Wang

China

Xujing Wang

USA

Hengliang Wang

China

Fengping Wang

China

Len Ward

Canada

John Warren

USA

Scott Weese

Canada

Grzegorz Wegrzyn

Poland

Francois-Xavier Weill

France

Jian-Fan Wen

China

Jeffrey Werner

USA

Silja Wessler

Austria

Nele Weyens

Belgium

Adrian Whatmore

UK

Paul Wichgers Schreur

Netherlands

Lothar H. Wieler

Germany

Odilia Wijburg

Australia

Gottfried Wilharm

Germany

Anne Willems

Belgium

Rob Willems

Netherlands

Erin Williams

Ireland

Laura Williams

USA

Brenda Anne Wilson

USA

Craig Winstanley

UK

Sebastian Winter

USA

Christoph Wittmann

Germany

Agnes Wold

Sweden

Alan Wolfe

USA

Annie Wong-Beringer

USA

Timothy Woo

UK

Andrew Wood

UK

Janet M. Wood

Canada

Lydia Wroblewski

USA

Ming-Shiang Wu

Taiwan

Jiunn-Jong Wu

Taiwan

Deng-Chyang Wu

Taiwan

Karina Xavier

Portugal

Chuanwu Xi

USA

Yechen Xiao

China

Defeng Xing

China

Meiying Xu

China

Jiru Xu

China

Jianping Xu

Canada

Xudong Xu

China

Javed Yakoob

Pakistan

Akio Yamada

Japan

Shouji Yamamoto

Japan

Yoshio Yamaoka

Japan

Yoshihisa Yamashita

Japan

Jie Yan

China

Kathy Yang

USA

Hongjiang Yang

China

Ming Yang

Canada

Ji Yang

Australia

Etienne Yergeau

Canada

Masahiro Yoneda

Japan

Yuko Yoshikawa

Japan

Chris Yost

Canada

Xue-Fu You

China

J Peter Young

UK

Ahmed Yousef

USA

Lijuan Yuan

USA

Jing Yuan

China

Sedigheh Zakeri

Iran

Fathiah Zakham

Yemen

Oscar Zaragoza

Spain

Egija Zaura

Netherlands

Andreas Zautner

Germany

Gianni Zehender

Italy

Mei Zeng

China

Ying Zhang

USA

Lian-Hui Zhang

Singapore

Jianzhong Zhang

China

Zhaojie Zhang

USA

Youfu Zhao

USA

Ning-Yi Zhou

China

Guoqiang Zhu

China

Weiming Zhu

China

Carl-Ulrich Zimmerman

Austria

Peter Zipfel

Germany

